# WNT16b promotes the proliferation and self‐renewal of human limbal epithelial stem/progenitor cells via activating the calcium/calcineurin A/NFATC2 pathway

**DOI:** 10.1111/cpr.13460

**Published:** 2023-03-27

**Authors:** Xichen Wan, Songjiao Zhao, Yiqin Dai, Jing Zhang, Yan Shen, Lan Gong, Qihua Le

**Affiliations:** ^1^ Department of Ophthalmology Eye, Ear, Nose and Throat Hospital of Fudan University Fudan China; ^2^ Department of Ophthalmology, Shanghai General Hospital Shanghai Jiao Tong University School of Medicine Shanghai China; ^3^ Research Centre Eye, Ear, Nose and Throat Hospital of Fudan University Fudan China; ^4^ Myopia Key Laboratory of Ministry of Health Eye, Ear, Nose and Throat Hospital of Fudan University Fudan China

## Abstract

Our previous finding revealed that WNT16b promoted the proliferation of human limbal epithelial stem cells (hLESCs) through a β‐catenin independent pathway. Here, we aimed to explore its underlying molecular mechanism and evaluate its potential in the treatment of limbal stem cell deficiency (LSCD). Based on the findings of mRNA‐sequencing, the expression of key molecules in WNT/calcineurin A/NFATC2 signalling pathway was investigated in WNT16b‐co‐incubated hLESCs and control hLESCs. An epithelial wound healing model was established on Wnt16b‐KO mice to confirm the regulatory effect of WNT16b in vivo. The therapeutic potential of WNT16b‐co‐incubated hLESCs was also evaluated in mice with LSCD. Our findings showed that WNT16b bound with Frizzled7, promoted the release of Ca^2+^ and activated calcineurin A and NFATC2. With the translocation of NFATC2 into cell nucleus and the activation of HDAC3, WDR5 and GCN5L2, the expression of H3K4me3, H3K14ac and H3K27ac in the promoter regions of FoxM1 and c‐MYC increased, which led to hLESC proliferation. The effect of the WNT16b/calcium/calcineurin A/NFATC2 pathway on LESC homeostasis maintenance and corneal epithelial repair was confirmed in Wnt16b‐KO mice. Moreover, WNT16b‐coincubated hLESCs could reconstruct a stable ocular surface and inhibit corneal neovascularization in mice with LSCD. In conclusion, WNT16b enhances the proliferation and maintains the stemness of hLESCs by activating the non‐canonical calcium/calcineurin A/NFATC2 pathway in vitro and in vivo*,* and accelerates corneal epithelial wound healing.

## Graphical abstract

After binding with FZD7, WNT16b causes the phosphorylation of PLCβ3, mobilizes the release of Ca^2+^ into the cytoplasm and activates calcineurin A. NFATC2 is dephosphorylated by calcineurin A and translocates into the cell nucleus and then binds to the promotors of c‐MYC and FoxM1. With the recruitment of GCN5L2, WDR5 and HDAC3, the enrichment of H3K27ac, H3K14ac and H3K4me3 occurs at the promoter area of FoxM1 and/or c‐MYC, which leads to the proliferation of hLESCs.

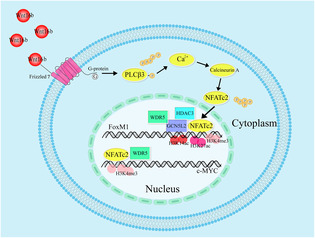

## 1 INTRODUCTION

Limbal epithelial stem/progenitor cells (LESCs) are essential for homeostasis maintenance and the regeneration of the corneal epithelium. A decrease in the population and/or dysfunction of LESCs usually leads to limbal stem cell deficiency (LSCD), which is characterized by conjunctivalization, persistent epithelial defects, ocular surface inflammation and scarring.^1^ Thus, restoration of functional LESCs is vital in the treatment of LSCD.

WNTs, a ligand family that consists of 19 proteins, play an important role in regulating the homeostasis maintenance of several adult stem cells, including bone marrow mesenchymal stem cells,^2^ neural stem cells, intestinal stem cells, hair follicle stem cells^3^ and LESCs.^4^, ^5^ In mammalian cells, WNT proteins participate in physiological processes via the canonical β‐catenin pathway and non‐canonical pathway.^6^ Non‐canonical WNT pathways include the WNT‐Ca^2+^ pathway, the WNT‐JNK pathway and other pathways activated through small GTPases.^7^, ^8^ WNT16, WNT6, WNT5a and WNT11 are the ligands of both the canonical β‐catenin pathway and non‐canonical WNT signalling pathways,^9^, ^10^, ^11^ while the other WNT proteins could only activate either of them. Recently, the interaction between the canonical and non‐canonical WNT signalling induced by WNT6 was reported to participate in the homeostasis regulation of human LESCs (hLESCs).^10^

The expression of WNT16b has mainly been found in the basal layer of the limbal epithelium,^12^ which is generally believed to be the location of hLESCs.^13^ Our previous study found that WNT16b enhanced the proliferation and self‐renewal of hLESCs, and this effect was not dependent on the canonical β‐catenin pathway.^9^ Among non‐canonical WNT pathways, the WNT‐Ca^2+^ pathway participates in the regulation of proliferation, self‐renewal and differentiation in various stem cells.^5^, ^14^, ^15^ WNT16 has been reported to promote the proliferation of osteoblasts^16^ by activating the WNT‐Ca^2+^ signalling pathway. After binding to Frizzled (FZD) receptors and other coreceptors, WNT16 activates phospholipase C (PLC) and causes calcium ion release from the endoplasmic reticulum, which further activates downstream PKC/CaMKII and/or calcineurin/NFAT pathways. Nevertheless, how the WNT‐Ca^2+^ signalling pathway mediated by WNT16b is involved in the homeostasis maintenance of hLESC remains unclear.

In this study, we investigated the role of the WNT‐Ca^2+^ signalling pathway on the regulation of proliferation and stemness maintenance of hLESCs. The results showed that WNT16b promoted the proliferation of hLESCs by activating the Ca^2+^/calcineurin A/NFATC2 pathway. Activated NFATC2 upregulated the expression of c‐MYC and FoxM1 via the recruitment of WDR5, HDAC3 and GCN5L2. Moreover, hLESCs treated with WNT16b could reconstruct a stable ocular surface and inhibit corneal neovascularization in an LSCD mice model. Our work reveals a novel mechanism of hLESC proliferation stimulated by WNT16b, which might be a potential tool to improve the quality of in vitro cultivated hLESCs for stem cell therapy.

## 2 RESULTS

### 2.1 The non‐canonical WNT‐Ca^2^ ^+^ pathway is involved in hLESC proliferation stimulated by WNT16b

**FIGURE 1:**
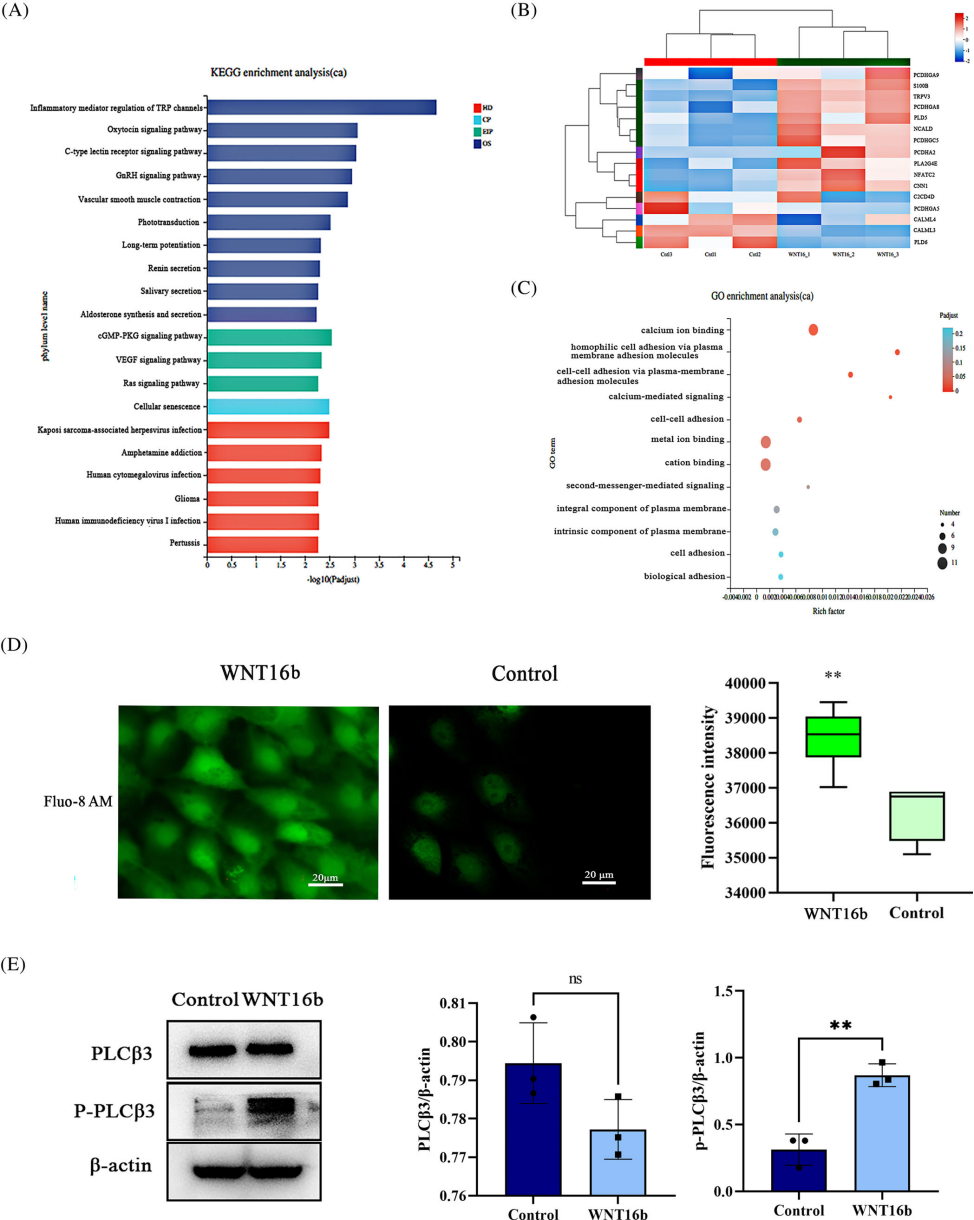
The non‐canonical WNT‐Ca^2+^ pathway is involved in human limbal epithelial stem cell (hLESC) proliferation induced by WNT16b. The mRNA‐seq assay showed that WNT/Ca^2+^ pathway‐related genes were significantly enriched after treatment with WNT16b, as shown by Kyoto Encyclopedia of Genes and Genomes (KEGG) (A), heatmap (B) and Gene Ontology (GO) (C) enrichment analyses. The fluorescence intensity of calcium was significantly higher in WNT16b‐treated hLESCs (*p* = 0.006) (D). Western blotting showed increased expression of pPLCβ3 after WNT16b treatment (*p* = 0.003), although the level of PLCβ3 was unchanged (E), confirming the mobilization of intracellular calcium. ** *p* < 0.01.

Our previous work showed that exogenous WNT16b (200 ng/mL) enhanced the proliferation of hLESCs (Figure S2A–E) in a β‐catenin‐independent manner.^9^ Based on our previous work, mRNA sequencing was performed to detect which non‐canonical WNT pathway was involved. According to the mRNA‐sequence assay (GEO database GSE180698), a total of 930 differentially expressed genes were found between the WNT16b‐treated group and the control group. We screened out non‐canonical WNT pathway‐related genes with Kyoto Encyclopedia of Genes and Genomes (KEGG) and Gene Ontology (GO) enrichment analyses. Molecules in the non‐canonical WNT‐Ca^2+^ pathway were found to be significantly enriched (Figure 1A–C), and the expression of NFATC2, a key transcription factor of the non‐canonical WNT/Ca^2+^/calcineurin/NFAT pathway, was significantly increased (|log2FC| = 1.28).

To identify whether the WNT/Ca^2+^ pathway was activated by WNT16b in hLESCs, we determined the changes in intracellular calcium using a fluorescence calcium probe. Treatment with WNT16b significantly increased the intracellular fluorescence intensity compared to that in the control group (*p* = 0.006, Figure 1D), suggesting that calcium might have been released from an intracellular pool by the stimulation of WNT16b. Moreover, the protein level of phosphorylated PLCβ3 (pPLCβ3), the active form of PLCβ3, also increased significantly (*p* = 0.003, Figure 1E). U73122, a potent inhibitor of pPLCβ3, significantly decreased the intracellular Ca^2+^ fluorescence intensity in WNT16b‐treated hLESCs, confirming that activated PLCβ3 plays a vital role in intracellular Ca^2+^ release (Figure S3A).

### 2.2 WNT16b actives the Ca^2+^/calcineurin A/NFATC2 signalling pathway

**FIGURE 2:**
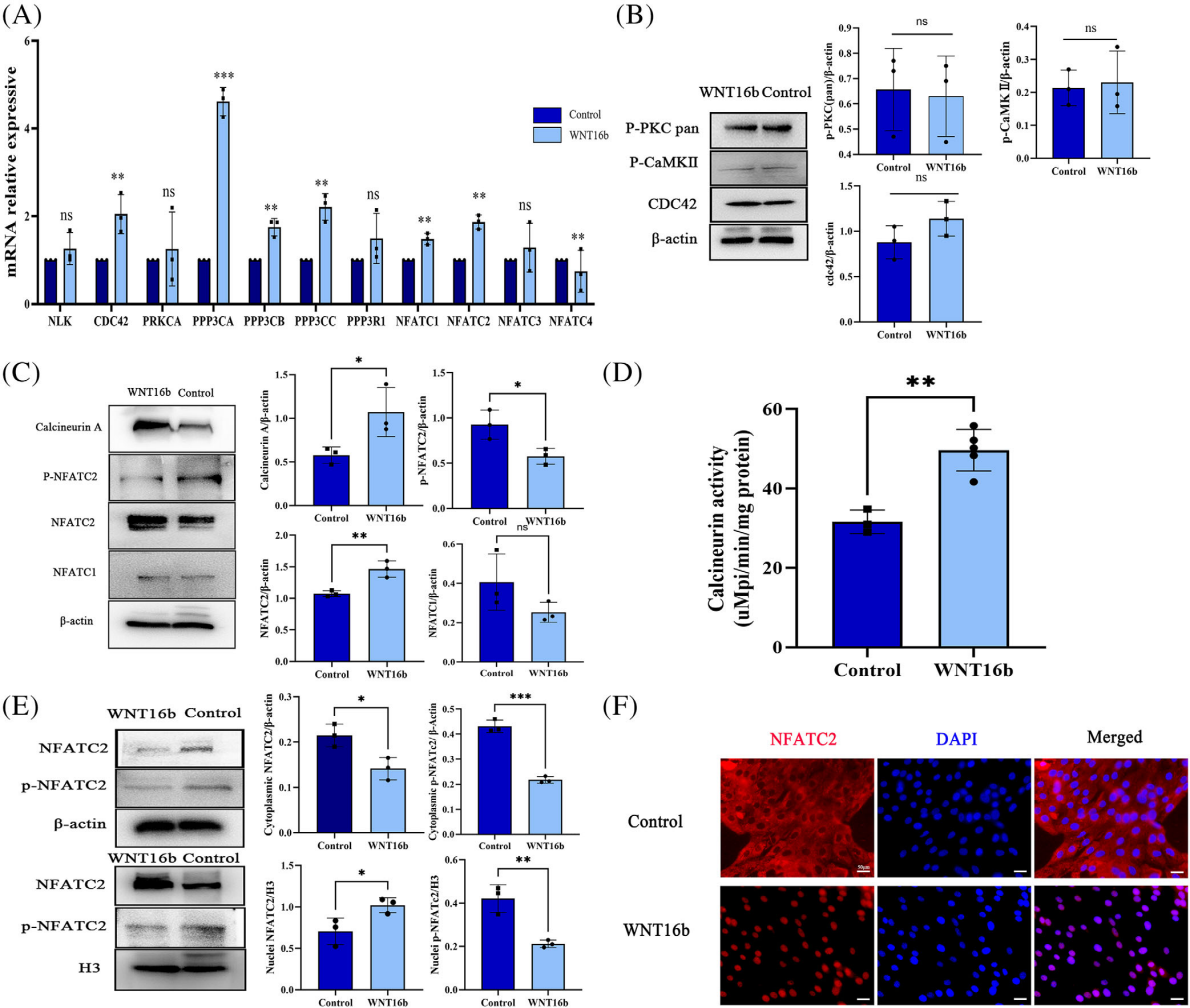
WNT16b activates the Ca^2+^/calcineurin A/NFATC2 signalling pathway. The mRNA levels of the genes encoding the catalytic subunits of calcineurin, *PPP3CA*, *PPP3CB* and *PPP3CC* and the isoforms of NFAT (*NFATC1* and *NFATC2*) were significantly increased in WNT16b‐treated human limbal epithelial stem cells (hLESCs) (*PPP3CA*: *p* < 0.001; *PPP3CB*: *p* = 0.003; *PPP3CC*: *p* = 0.002; *NFATC1: p* = 0.003; *NFATC2: p* = 0.001) (A). Nevertheless, WNT16b treatment did not affect the expression of *NLK* (*p* = 0.29), *PRKCA* (*p* = 0.63)*, PPP3R1* (*p* = 0.20) or *NFATC3* (*p* = 0.65). A reduction of only the mRNA level of *NFATC4* was found (*p* = 0.004), and the expression of CaMK2A was not detected. Although the expression of *CDC42* increased at the mRNA level (*p* = 0.002), it did not show a significant change at the protein level (*p* = 0.16) (B). The protein expression of other key molecules in the PKC/CaMKII pathway, such as p‐PKC pan, pCaMKII, was also unchanged (*p* = 0.84, 0.81, respectively). In contrast, WNT16b treatment significantly increased the protein levels of calcineurin A (*p* = 0.045) and NFATC2 (*p* = 0.008) (C) and enhanced the phosphatase activity of calcineurin A (*p* = 0.002) (D). However, it had no effect on NFATC1 (*p* = 0.15). Moreover, activated NFATC2 translocated from the cytoplasm into the cell nuclei after WNT16b treatment, as evidenced by Western blotting (E) and immunofluorescence staining (F). Scale bars: 50 μm. *p<0.05, ** *p* < 0.01, ***p < 0.001.

To clarify how the calcium pathway was activated by WNT16b, quantitative real‐time PCR (qRT–PCR) and Western blotting were performed to analyse the expression of several key molecules in the calcium pathway, including *PRKCA, CaMK2A, NLK, CDC42, PPP3CA* and its isoforms (*PPP3CB* and *PPP3CC*), and *NFATC2* and its isoforms (*NFATC1, NFATC3* and *NFATC4*). WNT16b treatment upregulated the mRNA levels of *NFATC2* (*p* = 0.001), *NFATC1* (*p* = 0.003) and *PPP3CA* (*p* < 0.001) and its isoforms (*PPP3CB*: *p* = 0.003, *PPP3CC*: *p* = 0.002; Figure 2A). Although the expression of *CDC42* increased at the mRNA level (*p* = 0.002), it did not show any significant changes at the protein level (*p* = 0.16, Figure 2B). None of the remaining molecules showed any significant changes at either the mRNA or protein level, except that the expression of *CaMK2A* was not detected. These findings indicate that the PKC/CaMKII pathway may not be involved in the proliferation of hLESCs activated by WNT16b and the mobilization of PLCβ3 and Ca^2+^.

Then, we tested whether the calcineurin/NFAT signalling pathway was activated after WNT16b treatment. The expression of calcineurin A was significantly higher in the WNT16b‐treated group than in the control group (*p* = 0.045, Figure 2C); an increase in phosphatase activity of calcineurin A was also observed (*p* = 0.002, Figure 2D). Although an overall elevated expression of NFATC2 was detected after the treatment with WNT16b (*p* = 0.008, Figure 2C), the changes of NFATC2 (active form) and phosphorylated NFATC2 (pNFATC2, inactive form) in the cell nuclei and cytoplasm were different. NFATC2 was mainly expressed in the nucleus rather than in the cytoplasm (nuclei: *p* = 0.04, cytoplasm: *p* = 0.02; Figure 2E), whereas the expression of pNFATC2 decreased significantly in both the cytoplasm and nucleus (nucleus: *p* = 0.005, cytoplasm: *p* < 0.001; Figure 2E). Immunofluorescence staining also confirmed the activation of NFATC2 and its translocation into the nuclei after treatment with WNT16b (Figure 2F). Together, these findings suggest that WNT16b treatment activates PLCβ_3_, promotes the release of Ca^2+^ into the cytoplasm, and then activates the calcineurin A/NFATC2 pathway in hLESCs.

### 2.3 WNT16b binds to FZD7 to activate the Ca^2+^/calcineurin A/NFATC2 pathway

**FIGURE 3:**
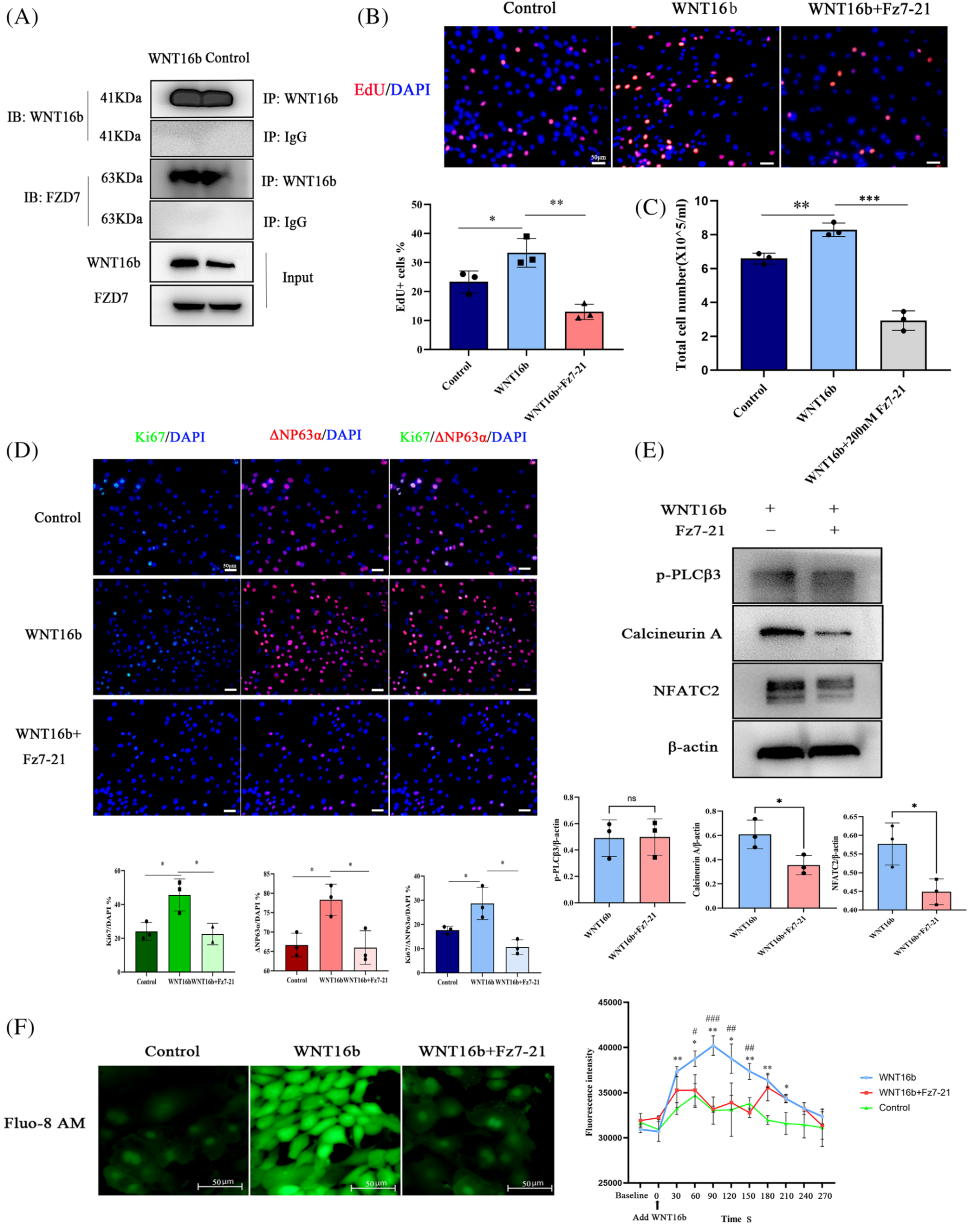
WNT16b binds to FZD7 to activate the downstream calcineurin A/NFATC2 signalling pathway. A coimmunoprecipitation (Co‐IP) assay (A) showed that WNT16b directly bound to FZD7. Blocking FZD7 with FZ7‐21 not only prohibited the effect of WNT16b on human limbal epithelial stem cell (hLESC) proliferation (B, C) and the expression of stemness biomarkers (D) (cell density: *p* < 0.001; EdU^+^ cell: *p* = 0.003; Ki67^+^ cell: *p* = 0.02; ΔNp63α^+^ cell: *p* = 0.02) but also downregulated the levels of calcineurin A (*p* = 0.04) and NFATC2 (*p* = 0.03) (E) and inhibited intracellular Ca^2+^ release (F). Only the expression of p‐PLCβ3 was not affected (*p* = 0.2). Scale bars: 50 μm. A~E: *p < 0.05, ** *p* < 0.01, ***p < 0.001. *in (F) stands for the comparison between WNT16b group and control, # stands for the comparison between WNT16b group and WNT16b+Fz7‐21 group. * and #: *p* < 0.05, ** and ﻿﻿##: *p* < 0.01, *** and ###: *p* < 0.001.

Next, we attempted to identify the receptors that bind to WNT16b to activate the Ca^2+^/calcineurin A/NFATC2 pathway. Among the FZD receptors expressed in mammals, only FZD7 is preferentially expressed in the limbus^17^ Therefore, coimmunoprecipitation (Co‐IP) was performed to determine the interaction between WNT16b and FZD7. It was found that more FZD7 could be precipitated in hLESCs treated with WNT16b (Figure 3A). A peptide antagonist of FZD7, Fz7‐21, which was added to the culture medium on Day 3 at a final concentration of 200 nM, suppressed hLESC proliferation induced by WNT16b (Figure 3B, C, Figure S3B) and downregulated the expression of the stemness biomarker ΔNp63α (Figure 3D). Moreover, after Fz7‐21 treatment, the release of Ca^2+^ induced by WNT16b was inhibited, along with decreased expression of calcineurin A and NFATC2 (Figure 3E,F). These findings suggest that WNT16b binds to the FZD7 receptor to activate the downstream Ca^2+^/calcineurin A/NFATC2 signalling pathway.

### 2.4 Inhibition of the calcineurin A/NFATC2 signalling pathway attenuates the WNT16b‐stimulated proliferation of hLESCs

**FIGURE 4:**
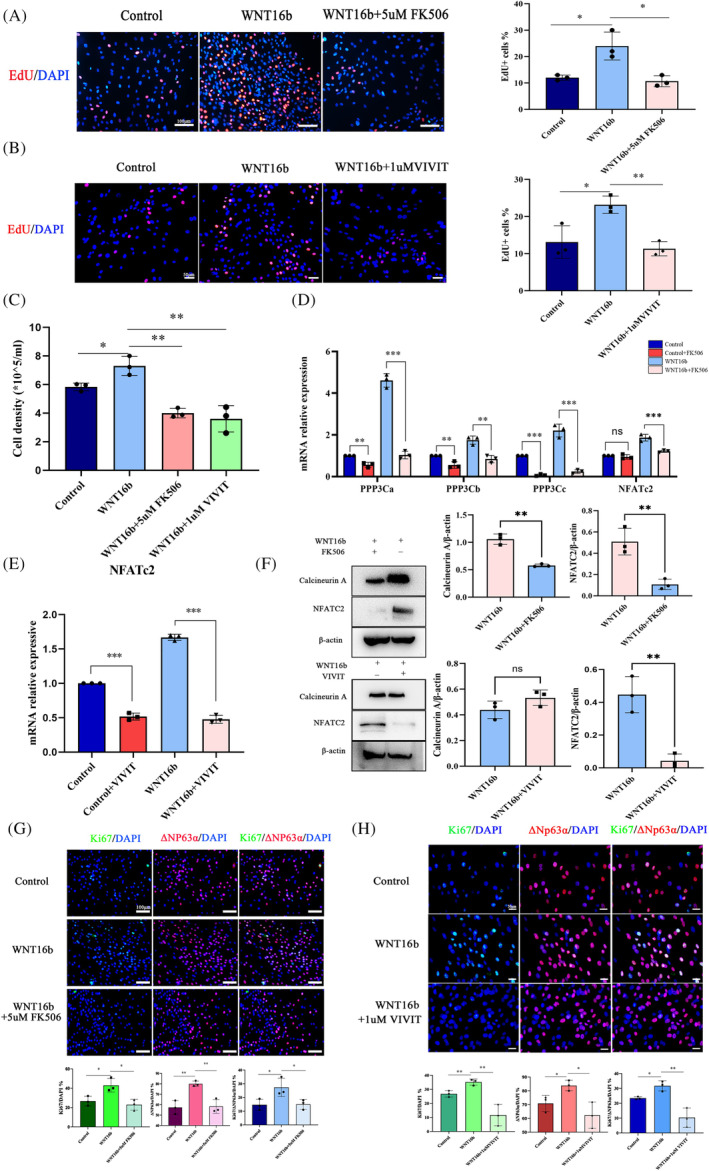
Inhibition of the calcineurin A/NFATC2 signalling pathway attenuates the proliferation of human limbal epithelial stem cells (hLESCs) induced by WNT16b. EdU assays (A, B) showed that both 5 μM FK506 (A) and 1 μM VIVIT (B) attenuated hLESC proliferation induced by WNT16b (*p* = 0.02 and 0.001, respectively), which was further confirmed by a decrease in cell density (*p* = 0.002 and 0.005, respectively; C). RT–PCR (D, E) showed that the expression of *PPP3CA* (*p* < 0.001)*, PPP3CB* (*p* = 0.004), *PPP3CC* (*p* < 0.001) and *NFATC2* (*p* < 0.001) decreased with FK506 treatment (D), whereas VIVIT caused a reduced level of only NFATC2 (*p* < 0.001; E). Accordingly, Western blotting (F) revealed that FK506 inhibited the expression of both calcineurin A and NFATC2 (*p* = 0.001 and 0.007, respectively), while VIVIT only prohibited the expression of NFATC2 (calcineurin A: *p* = 0.15, NFATC2: *p* = 0.004). In addition, the increased expression of ΔNp63α and Ki67 in WNT16b‐treated hLESCs was suppressed by treatment with either FK506 (*p* = 0.05 and 0.02, respectively; G) or VIVIT (*p* = 0.02 and 0.007, respectively; H). Scale bars: 50 μm (B, H); 100 μm (A, G). *p < 0.05, ** *p* < 0.01, ***p < 0.001.

According to our preliminary study (Figure S3C,D), FK506, a specific inhibitor of calcineurin, and VIVIT, a selective NFAT inhibitor, were used to verify the involvement of the calcineurin A/NFATC2 pathway in the proliferation of hLESCs induced by WNT16b. The coincubation of either 5 μM FK506 or 1 μM VIVIT with hLESCs from Day 3 inhibited the effect of WNT16b on cell proliferation, as evidenced by a lower percentage of EdU^+^ cells (FK506: *p* = 0.02, VIVIT: *p* = 0.001; Figure 4A,B) and a decrease in cell density (FK506: *p* = 0.002, VIVIT: *p* = 0.005; Figure 4C).

Moreover, the mRNA levels of *NFATC2* and all isoforms of *PPP3C* decreased with FK506 treatment regardless of the presence of WNT16b (*PPP3CA*: *p* < 0.001, *PPP3CB*: *p* = 0.004, *PPP3CC*: *p* < 0.001, *NFATC2: p* < 0.001, Figure 4D). Accordingly, FK506 significantly inhibited the protein expression of both calcineurin A and NFATC2 (calcineurin A: *p* = 0.001, NFATC2: *p* = 0.007; Figure 4F). VIVIT inhibited the expression of NFATC2 at both the mRNA level (*p* < 0.001, Figure 4E) and protein level (*p* = 0.004, Figure 4F) but had no effect on calcineurin A, confirming that NFATC2 was the downstream molecule of calcineurin A. Immunofluorescence staining showed that WNT16b promoted the expression of the cell proliferation marker Ki67 and the putative hLESC marker ΔNp63α. Nevertheless, this effect was significantly inhibited by treatment with either FK506 or VIVIT (Figure 4G,H). The combination of these findings suggests that blocking the calcineurin A/NFATC2 signalling pathway suppresses the WNT16b‐stimulated proliferation of hLESCs.

### 2.5 WNT16b modulates the expression levels and epigenetic modification of c‐MYC and FoxM1

**FIGURE 5:**
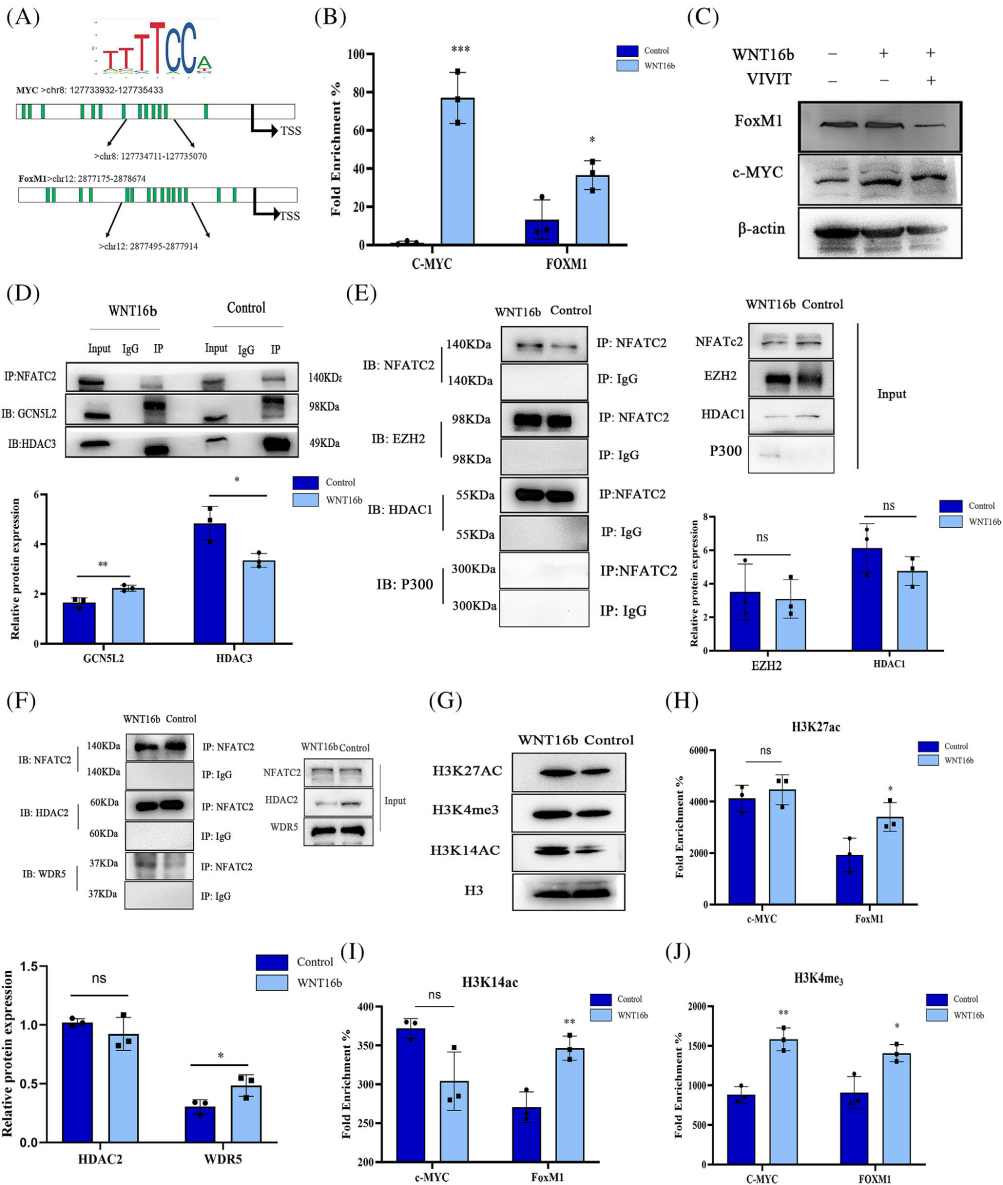
WNT16b modulates the epigenetic modifications on *c‐MYC* and *FoxM1*. Schematic diagram (A) showed the locations of the NFATC2 binding sites in the *c‐MYC* and *FoxM1* promoters. The sequences at the most frequent binding sites were used for the ChIP–qPCR assay. WNT16b induced an enrichment of NFATC2 at the promoters of *c‐MYC* and *FoxM1* (*p* < 0.001 and *p* = 0.034, respectively; B). The inhibition of NFATC2 with VIVIT treatment prohibited the expression of FoxM1 and c‐MYC (C). Coimmunoprecipitation (Co‐IP) assays (D‐F) showed upregulation of GCN5L2 (*p* = 0.005; D) and WDR5 (*p* = 0.01; F) and downregulation of HDAC3 (*p* = 0.03; D) after WNT16b treatment. Nevertheless, HDAC1, HDAC2 and EZH2 did not have significant changes compared with the control group (E). Elevated levels of H3K27ac, H3K14ac and H3K4me3 were also found in the nuclei of WNT16b‐treated hLESCs (G). ChIP–qPCR (H–J) revealed the enrichment of H3K4me3 (J), H3K14ac (I) and H3K27ac (H) at the promoter loci of *FoxM1* (H3K4me3: *p* = 0.021, H3K14ac: *p* = 0.006; H3K27ac: *p* = 0.04) and the enrichment of H3K4me3 in the promoter regions of *c‐MYC* (*p* = 0.002; J). *p < 0.05, ** *p* < 0.01, ***p < 0.001

To investigate the underlying molecular mechanism of WNT16b on hLESCs, chromatin immunoprecipitation (ChIP–qPCR) was performed to explore the target genes of NFATC2. To the best of our knowledge, *c‐MYC* and *FoxM1* are genes related to cell proliferation and have been identified as target genes of the WNT signalling pathway.^18^, ^19^ Therefore, we scanned the proximal promotor region (1.5 kb upstream from TSS) of *c‐MYC* and *FoxM1* and predicted the binding sites using JASPAR software (http://jaspar.genereg.net) (Figure 5A). Thirteen and fifteen binding sites of NFATC2 were found in the promoters of *c‐MYC* and *FoxM1*, respectively. With WNT16b treatment and the consequently increased expression of NFATC2, the promoter regions of both *c‐MYC* (chr8: 127734711–127735070) and *FoxM1* (chr12: 2877495–2877914) exhibited significant enrichment (*c‐MYC*: *p* < 0.001; *FoxM1*: *p* = 0.034) (Figure 5B). Moreover, this effect was significantly inhibited by the addition of VIVIT (Figure 5C).

Then, the levels of consensus epigenetic marks of active transcription were tested. The expression levels of trimethylated lysine 4 of histone 3 (H3K4me3), acetylated lysine 14 of histone (H3K14ac) and acetylated lysine 27 of histone 3 (H3K27ac) were higher in the nuclei of WNT16b‐treated cells (Figure 5G). Intriguingly, H3K4me3 was found to be enriched in both the *c‐MYC* (*p* = 0.002) and *FoxM1* (*p* = 0.021) promoter loci, while the enrichment of H3K14ac and H3K27ac markedly increased only in the promoter regions of *FoxM1* (H3K14ac: *p* = 0.006; H3K27ac: *p* = 0.04) rather than that of *c‐MYC* (Figure 5H–J). The interactions of NFATC2 with histone deacetylases (HDACs), histone acetylase P300, histone deacetylase GCN5L2 and methyltransferase WDR5 were also investigated. Elevated expression of GCN5L2 (*p* = 0.005) and WDR5 (*p* = 0.01) and decreased expression of HDAC3 (*p* = 0.03) were found in WNT16b‐treated hLESCs. P300 did not interact with NFATC2 (Figure 5D–F). These data suggest that after activation and translocation into cell nuclei, NFATC2 binds to the promoters of *c‐MYC* and *FoxM1* directly and regulates histone modification enzymes, including HDAC3, GGCN5L2 and WDR5. Then, H3K14ac, H3K27ac and/or H3K4me3 are enriched in the promoters of *c‐MYC* and *FoxM1*, which subsequently leads to the proliferation of hLESCs.

### 2.6 Exogenous WNT16b promotes corneal wound healing in Wnt16b‐KO mice

**FIGURE 6:**
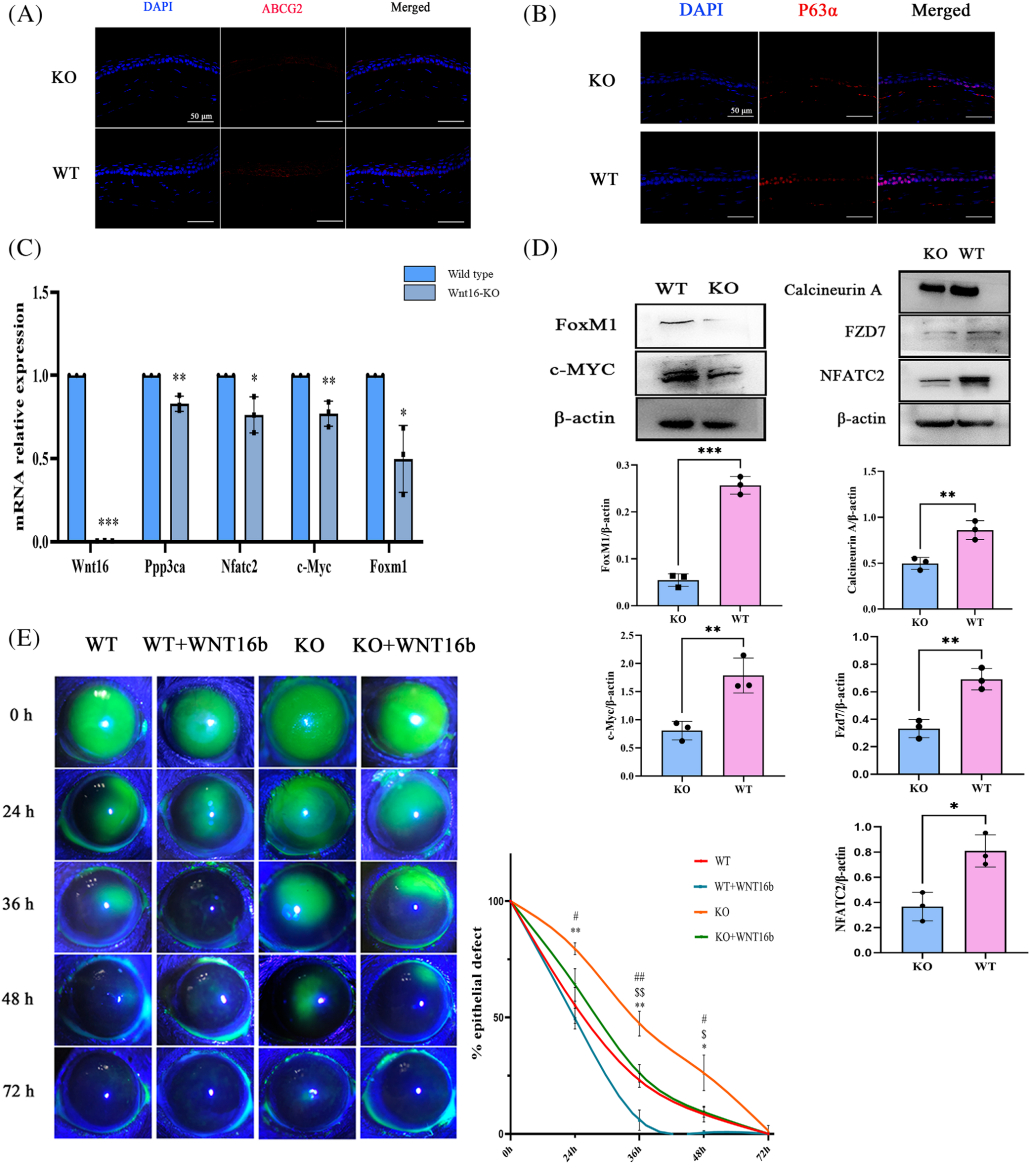
WNT16b promotes corneal wound healing in Wnt16b‐KO mice. The expression of P63α and ABCG2 in the limbal epithelium (A–B) was significantly decreased in Wnt16b‐KO mice. Moreover, both qRT–PCR (C) and Western blotting (D) showed downregulated expression of FZD7, calcineurin A, NFATC2, c‐MYC and FoxM1 (qRT–PCR: *Ppp3ca*: *p* = 0.002, *Nfatc2*: *p* = 0.02, *c‐Myc*: *p* = 0.006, *Foxm1*: *p* = 0.01; WB: calcineurin A: *p* = 0.007, NFATC2: *p* = 0.01, FZD7: *p* = 0.004; FoxM1: *p* = 0.0001, c‐MYC: *p* = 0.009). Evaluation of the epithelial defect sizes showed that Wnt16b‐KO mice had a slower epithelial healing rate than WT mice (24 h: *p* = 0.007, 36 h: *p* = 0.002, 48 h: *p* = 0.021). Topical application of WNT16b significantly accelerated epithelial healing in both WT mice (36 h: *p* = 0.005, 48 h: *p* = 0.016) and Wnt16b‐KO mice (24 h: *p* = 0.02, 36 h: *p* = 0.005, 48 h: *p* = 0.022) (E). KO: WNT16b‐knockout mice; WT: wild‐type mice. *,# and $ stand for the subgroup comparison between WT and KO, KO and KO+WNT16b, and WT and WT+WNT16b, respectively. *,#,$: *p* < 0.05, **,##, $$: *p* < 0.01, ***: *p* < 0.001.

The expression of P63α and ABCG2 was significantly lower in the limbal epithelium of Wnt16b‐KO mice (Figure 6A,B). Moreover, the expression of FZD7, calcineurin A, NFATC2, *c‐MYC* and *FOXM1* was significantly downregulated in Wnt16b‐KO mice (Figure 6C,D). These in vivo findings were consistent with what we found in the in vitro experiments.

The potency of WNT16b to promote the proliferation of LESCs in vivo was also evaluated, as shown in Figure 6E. The size of the epithelial defect was evaluated at 24, 36, 48 and 72 h after the epithelial wound model was established. Compared with the WT group, Wnt16b‐KO mice had the slowest epithelial healing rate (24 h: *p* = 0.007, 36 h: *p* = 0.002, 48 h: *p* = 0.021). Treatment with WNT16b significantly accelerated epithelial healing in both WT mice (36 h: *p* = 0.005, 48 h: *p* = 0.016) and Wnt16b‐KO mice (24 h: *p* = 0.02, 36 h: *p* = 0.005, 48 h: *p* = 0.022). These findings suggest that *Wnt16b* deficiency causes delayed corneal epithelial wound healing and that exogenous WNT16b accelerates wound healing by promoting LESC proliferation.

### 2.7 WNT16b‐treated hLESCs reconstruct a stable ocular surface and inhibit corneal neovascularization in LSCD eyes

In total LSCD mice eyes, persist epithelial defect was present even on 60 days after alkali burn, along with corneal neovascularization and stromal opacity, which indicated a complete destruction of functional LESCs (Figure S4A,B). The histopathological findings showed that only 1–2 layers of disorganized oedematous epithelial cells were observed on the corneas of LSCD eyes. Moreover, the corneal stroma was severely thickening with neovascularization and inflammatory infiltration (Figure S4C).

**FIGURE 7:**
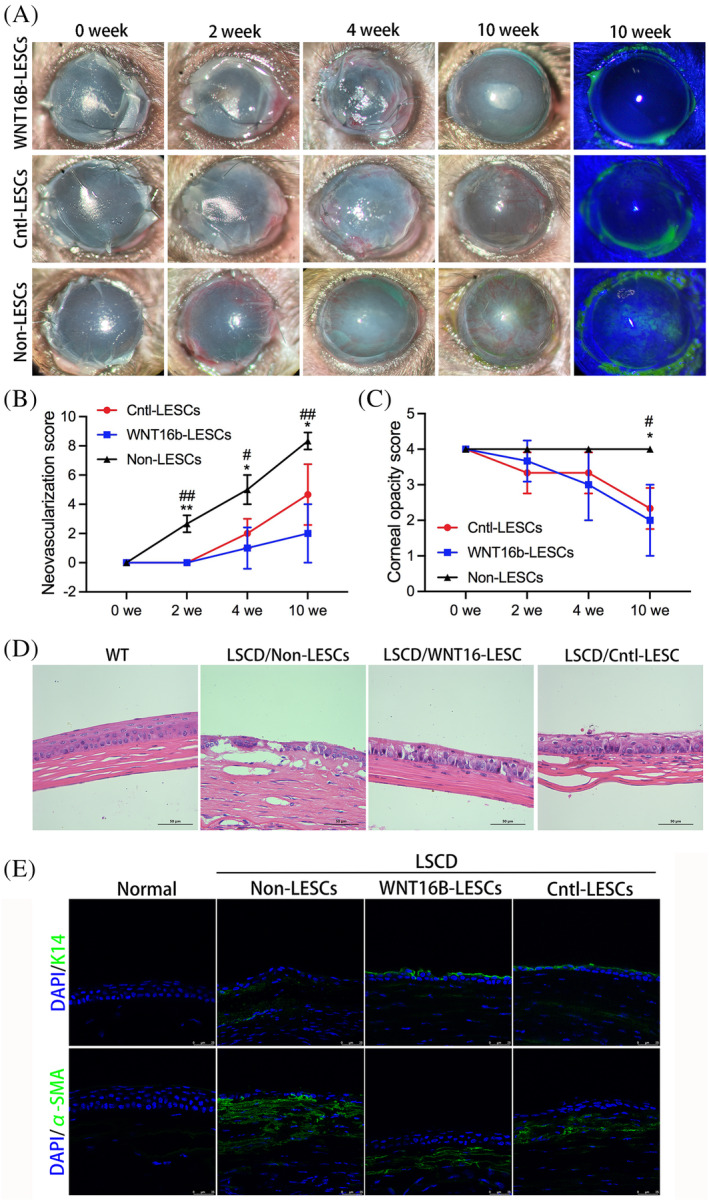
Evaluation of ocular surface reconstruction in total limbal stem cell deficiency (LSCD) eyes treated with or without human limbal epithelial stem cells (hLESCs) transplantation. Representative slitlamp images of the ocular surface on 0, 2, 4 and 10 weeks (A) revealed a complete epithelium recovery after the treatment of WNT16b‐hLESCs (*n* = 6) and Cntl‐hLESCs (*n* = 6) for 10 weeks. Fluorescein staining was present in eyes without hLESCs transplantation (*n* = 6). Among three groups, the lowest neovascularization score was found in WNT16b‐hLESCs‐treated group at 10 weeks after the treatment (*p* = 0.006 and 0.042; B). The corneal opacity scores of WNT16b‐hLESCs‐ and Cntl‐hLESCs‐treated groups were similar, both of which were significantly lower than that in Non‐hLESCs group (*p* = 0.026 and 0.017; C). The corneal epithelial cells in WNT16b‐hLESCs‐treated group were recovered to 4–5 layers but slightly irregularly arranged as evidenced by histopathological examination (D). Moreover, the stroma oedema completely vanished and little neovascularization was observed in WNT16b‐hLESCs‐treated group. Compared to WNT16b‐hLESCs‐treated group, stromal neovascularization was still found in Cntl‐hLESCs‐treated group. Immunofluorescence staining of K14 and α‐SMA (E) showed that human derived K14^+^ cells were identified on the corneal surface of WNT16b‐hLESCs and Cntl‐hLESCs‐treated groups. No human K14^+^ cells were found in either LSCD eyes without hLESCs transplantation or normal mice eyes. The expression level of α‐SMA in WNT16b‐hLESCs and Cntl‐hLESCs‐treated groups was significantly lower than that in Non‐LESCs treated group. *The comparison between Cntl‐hLESCs and Non‐hLESCs‐treated groups. *p < 0.05, **p < 0.01. #, the comparison between WNT16b‐hLESCs‐ and Non‐hLESCs‐treated groups. #*p*<0.05，##*p*<0.01.

Sixty days after alkali injury, the LSCD eyes were randomly divided into three groups and treated with or without hLESCs transplantation. After 10 weeks follow‐up, fluorescein staining was still present in eyes without hLESCs transplantation, indicating the presence of corneal epithelial defect, while the corneal epithelium was completely recovered in WNT16b‐hLESCs and Cntl‐hLESCs‐treated groups (Figure 7A). Among three groups, the lowest neovascularization score was found in WNT16b‐hLESCs‐treated group, which was significantly lower than those in Non‐hLESCs‐treated group (*p* = 0.006) and Cntl‐hLESCs‐treated group (*p* = 0.042) (Figure 7B). Although corneal opacity was partially alleviated in WNT16b‐hLESCs‐ and Cntl‐hLESCs‐treated groups compared to Non‐hLESCs‐treated group, the stroma scar could not completely vanish after hLESC transplantation (Figure 7A, C). The histopathological findings confirmed that the corneal epithelium in WNT16b‐hLESCs‐treated group was composed of 4–5 layers of epithelial cells, along with the diminishment of stromal oedema and little stromal neovascularization. Nevertheless, the recovered epithelial cells were slightly irregularly arranged due to the incomplete reconstruction of intercellular tight junctions (Figure 7D). To identify the cell engraftment after hLESCs transplantation, immunostaining was used to detect the expression of human K14. It was found that K14^+^ cells were visible on the corneal surface of WNT16b‐hLESCs‐ and Cntl‐hLESCs‐treated groups. The expression of α‐smooth muscle actin (α‐SMA) was also detected to evaluate the formation of stromal scar, and it turned out that α‐SMA was found in all groups although its expression level in WNT16b‐hLESCs and Cntl‐hLESCs‐treated groups were significantly lower than that in Non‐hLESCs‐treated group (Figure 7E).

## 3 DISCUSSION

**FIGURE 8:**
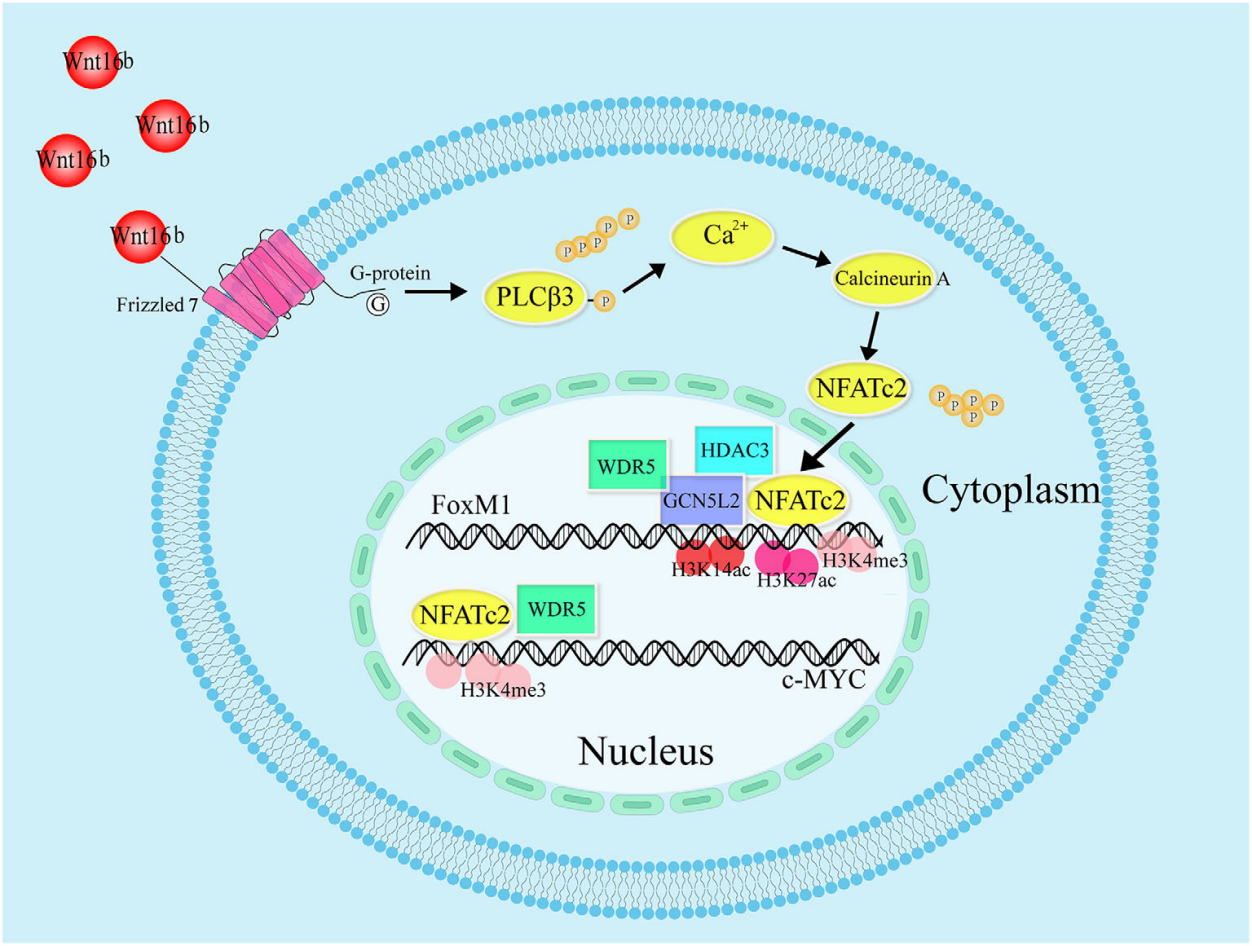
Schematic diagram of the molecular mechanism of the WNT16b/FZD7/calcineurin A/NFATC2 pathway in WNT16b‐treated hLESCs. With binding to FZD7, WNT16b causes the phosphorylation of PLCβ3, mobilizes the release of Ca^2+^ into the cytoplasm and activates calcineurin A. NFATC2 is dephosphorylated by calcineurin A and translocates into the cell nucleus and then binds to the promotors of c‐MYC and FoxM1. With the recruitment of GCN5L2, WDR5 and HDAC3, the enrichment of H3K27ac, H3K14ac and H3K4me3 occurs at the promoter area of FoxM1 and/or c‐MYC, which leads to the proliferation of hLESCs.

WNT16b has been confirmed to play a functional and critical role in promoting proliferation and maintaining the stemness of hLESCs. However, its molecular mechanism has not been fully elucidated. The current study reveals that WNT16b promotes the proliferation and stemness maintenance of hLESCs by binding to FZD7 and activating the Ca^2+^/calcineurin A/NFATC2 signalling pathway, which was confirmed by both in vitro and in vivo studies (Figure 8). Our study is the first to report the involvement of the WNT/Ca^2+^ signalling pathway in the maintenance of homeostasis in hLESCs.

FZD7, an important member of the FZD family, plays a critical role in stem cell homeostasis via interactions with WNT ligands and the regulation of intracellular signalling molecules, including Ca^2+^.^20^, ^21^ Flanagan et al. found that FZD7 was necessary to maintain the self‐renewal of Lgr5^+^ stem cells.^20^ The FZD7 antagonist Fz7‐21 was reported to impair the WNT pathway in intestinal stem cells.^22^ More importantly, FZD7 was the only FZD receptor that colocalized with hLESCs and was found to be indispensable to maintain the undifferentiated status of hLESCs.^17^ The current study confirmed the importance of FZD7 in the activation of the WNT‐Ca^2+^ signalling pathway and self‐renewal of hLESCs.

The WNT‐Ca^2+^ signalling pathway plays an important role in embryonic development, tumour regression, stem cell self‐renewal and the inflammatory response.^23^ The PKC/CaMKII pathway and calcineurin A/NFAT pathway are the most common downstream signalling pathways that are activated after the mobilization and release of Ca^2+^. The current study showed that only the Ca^2+^/calcineurin A/NFATC2 pathway was activated in the WNT16b‐stimulated proliferation of hLESCs. NFATC2, an important transcription factor that was first discovered in T cells,^24^ is dephosphorylated by calcineurin A and preferentially binds to the promoter or enhancer regions of target genes by the TTCCC consensus sequence.^25^ The involvement of the Ca^2+^/calcineurin/NFAT signalling pathway and the activation of *c‐MYC* and *FoxM1* has been reported to participate and regulate cellular proliferation in various types of cells.^26^, ^27^, ^28^, ^29^

Epigenetic modification plays an important role in adult stem cells. Previous studies have revealed that NFAT isoforms could recruit methyltransferase Suv39H1, Ezh2, the histone acetylase P300/CBP, and histone deacetylases class I, II and III (HDACs 1, 2, 3).^25^, ^28^, ^30^ Nevertheless, the current study identified enrichment of GCN5L2 and WDR5 rather than P300 after the activation of NFATC2 in hLESCs, along with decreased HDAC3 expression. Consequently, epigenetic modifications, including H3K14ac and H3K27ac in the promoter region of FoxM1 and H3K4 trimethylation in the promoter region of both c‐MYC and FoxM1, were found. H3K14ac, H3K27ac and H3K4me3 participate in various physiological and pathological processes, such as retinal development,^31^ leukaemia stem cell maintenance,^32^ and embryonic stem cell differentiation.^33^

*FoxM1* is involved in self‐renewal and cell proliferation in human epithelial stem/progenitor cells^34^ and promotes the maintenance of stem cell properties in cancer stem cells.^35^ Moreover, it has been shown to bind to β‐catenin directly and participate in the canonical WNT signalling pathway.^18^ Our study is the first to report its involvement in the non‐canonical WNT/Ca^2+^ signalling pathway. *c‐MYC* is also a target gene of the WNT signalling pathway, which is involved in cell proliferation, cell cycle regulation,^36^ stem cell fate determination^37^ and the maintenance of stem cell properties.^38^ It was reported that NFATC2 could upregulate the expression of c‐MYC by binding to the TIE element, a special sequence within the proximal promoter of *c‐MYC*.^29^ The current study revealed a novel binding region on the *c‐MYC* gene, −723 to −364 from the TSS (chr8: 127734711–127735070), which merits further investigation.

The current study showed that the transplantation of WNT16b‐coincubated hLESCs could safely reconstruct a stable ocular surface in eyes with total LSCD, which might be better than traditionally cultivated hLESC regarding the inhibition of stromal neovascularization.^39^, ^40^ However, neither the transplantation of WNT16b‐treated hLESCs nor routine hLESCs transplantation could completely remove stromal scars, indicating that a penetrating keratoplasty is usually necessary for severe LSCD eyes to improve the vision after the function of LESCs is restored and the ocular surface homeostasis is reconstructed.

In conclusion, WNT16b promotes the proliferation of hLESCs and accelerates corneal epithelial wound healing by binding to FZD7 and activating the Ca^2+^/calcineurin A/NFATC2 signalling pathway. WNT16b might be a potential tool to maintain the self‐renewal ability of hLESCs cultured in vitro and improve the quality of cultured cell sheets for hLESC transplantation.

## 4 METHODS

### 4.1 Primary human limbal epithelial cell culture

Cadaveric human corneoscleral tissues that were stored in Optisol at 4°C for less than 5 days were obtained from an eye bank. The ages of the donors ranged from 20 to 70 years old, and all tissues used for cell culture did not meet the criteria for clinical use. Excessive sclera, conjunctival, iris and trabecular meshwork were removed carefully from the corneoscleral rim after a thorough wash in phosphate‐buffered saline (PBS, HyClone) with 100 U/mL penicillin, 100 U/mL streptomycin and 1.25 μg/mL amphotericin B for three times. Then, the corneoscleral rim was cut into small 1 mm × 1 mm pieces with the epithelial side facing towards the bottom of the dish to facilitate cell outgrowth. DMEM/F12 growth medium (HyClone) supplemented with 1% N‐2 (Gibco), 5 ng/μL EGF (Sigma–Aldrich), 0.5% dimethyl sulfoxide (Sigma–Aldrich), 10% foetal bovine serum (Gibco), 100 U/mL penicillin and 100 mg/mL streptomycin (Gibco) were added. Recombinant human WNT16b (rhWnt16b, R&D systems) was added to the medium to achieve a final concentration of 200 ng/mL from Day 3. The specific inhibitors used in inhibitory experiments were added also on Day 3 such as FK506, VIVIT and Fz7‐21 (MedChemExpress, final concentrations shown in the Results Section). Cells were incubated at 37°C with /5% CO_2_ for approximately 14 days, and the growth medium was refreshed every 3 days (Figure S1A).

### 4.2 Assessment of cell proliferation

An EdU cell proliferation kit (Beyotime Biotechnology, China) was used to assess cell proliferation when cell growth was in logarithmic phase (Day 10). According to the manufacturer's instructions, the EdU solution was added to the culture medium to achieve a final concentration of 10 μM followed by coincubation for 6 h. After that, the cells were treated with 4% paraformaldehyde for 15 min and 0.3% Triton for 10 min. Then, the click relative solution was added to cross‐link EdU with the fluorescent azide, which can be detected under a fluorescence microscope. The percentage of EdU+ cells was determined. In addition, hLESCs cultured in a 24‐well culture plate were harvested on Day 12, and the cell number was counted to calculate the cell density.

### 4.3 Quantitative RT–PCR

Cells were harvested on Day 14 (Figure S1A) and total RNA was extracted using TRIzol reagent (Invitrogen) according to the manufacturer's protocol. The PrimeScript™ RT reagent Kit with gDNA Eraser (TaKaRa) was used for reverse transcription of cDNA. qRT–PCR was performed with a QIAantiNova SYBR Green PCR kit (Qiagen) with a protocol of 2 min of initial activation at 95°C, 40 cycles of 5 s of denaturation at 95 °C and 20 s of annealing and extension at 60°C. mRNA expression levels were determined using the 2^−ΔΔCt^ method, in which the target gene was normalized to β‐actin to calculate the relative mRNA fold change. Three replicates were performed, and the data are shown as the mean ± SD. The sequences of the target gene‐specific primers are shown in Table S1.

### 4.4 Immunofluorescence staining

hLESCs cultured on Matrigel‐coated 24‐chamber slides for 12 days (Figure S1A) were fixed with 4% paraformaldehyde for 15 min. Mice corneal tissues were fixed with 4% paraformaldehyde, dehydrated in graded sucrose, embedded in an optimum‐cutting‐temperature compound (SAKURA), and persevered at −80 °C. A freezing microtome (Leica) was used to cut the embedded tissue into 10‐μm‐thick sections. Cultured‐hLESCs and mice cornea tissues were washed with PBS and permeabilized with 0.3% Triton X‐100 (Sigma–Aldrich). After being blocked in 3% bovine serum albumin (Sigma–Aldrich) in PBS for 30 min at room temperature, hLESCs and mice corneal tissues were incubated with primary antibodies at 4°C overnight. Then, the cells and sections were washed with PBS three times and incubated with secondary antibodies at room temperature for 1 h. The nuclei were labelled with Hoechst 33258 (Invitrogen, 1:2000) and mounted in 50% glycerinum. Images were acquired with a fluorescence microscope (Zeiss, Scope. A1, Germany). Antibody information is listed in Table S2.

### 4.5 Western blotting

hLESCs were harvested on Day 14 and washed once with PBS, and total protein was isolated using RIPA buffer (Beyotime Biotechnology, China). The nuclei and cytoplastic protein were extracted with a NE‐PER™ Nuclear and Cytoplasmic Extraction Reagents kit (Thermo Fisher Scientific, USA). The protein concentration was detected with a BCA Protein Assay kit (Beyotime Biotechnology, China). Protein was separated by 8%–12.5% SDS‐polyacrylamide gel electrophoresis (Beyotime Biotechnology, China) and transferred to a nitrocellulose membrane (PVDF, Millipore) according to standard protocols. The membranes were blocked with 5% non‐fat powdered milk and incubated with primary antibodies at 4°C overnight. After a thorough wash with Tris‐buffered saline with Tween (TBST), HRP‐conjugated secondary antibodies were added for incubation with the membranes at room temperature for 1 h. β‐Actin and histone 3 were used as internal references for total protein and nucleoprotein, respectively. The protein expression levels were semiquantified with ImageJ software (NIH, Washington, DC). Antibody information is listed in Table S2.

### 4.6 Intracellular calcium imaging

Fz7‐21, U73122 and U73433 were added in the culture medium on Day 9. After 3‐day treatment, a Fluo‐8 calcium flux assay kit (Abcam) was performed to detect intracellular calcium mobilization (Figure S1B). According to the manufacturer's protocol, 10 μM Fluo‐8 AM, an intracellular calcium ion fluorescent probe, was added to 2 × 10^4^ cells, incubated at 37°C for 30 min and then cooled at room temperature for another 30 min. The fluorescence intensity of cytoplasm calcium was measured with a fluorescence microplate reader at Ex/Em = 490/525 nm as the baseline. Then exogenous WNT16b was added in the culture medium, and the fluorescence intensity of calcium release was recorded with an interval of every 30 s. The fluorescence microscopic images of intracellular calcium were captured at 2 min after exogenous WNT16b was added.

### 4.7 Calcineurin activity assessment

Calcineurin phosphatase activity in hLESCs was evaluated on Day 14 by the application of a cellular calcineurin activity kit (Genmed Scientifics Inc.). Briefly, cell lysate was prepared and incubated with RII phosphopeptide substrate and assay buffer at 30°C for 10 min. Malachite green reagent was added to estimate the release of phosphate. The absorbance was measured with a microplate reader (TECAN) at 660 nm at 37°C. The calcineurin phosphatase activity was calculated as μM pi released/min/mg protein.

### 4.8 Chromatin immunoprecipitation–qPCR

After the cells were collected on Day 14, ChIP‐qPCR was performed to identify the target genes binding with the transcription factor NFAT and the amount of histone H3 modification. A high‐sensitivity ChIP Kit (Abcam) was used for the ChIP assay. Briefly, 1% formaldehyde was added to the medium to cross‐link the transcription factors to chromatin for 10 min at room temperature, and then formaldehyde was inactivated with the addition of 125 mM glycine. Sonication was carried out for chromatin shearing, and chromatin containing DNA fragments between 100 and 700 bp in length were used for immunoprecipitation. Normal IgG and polymerase II provided in the kit were used as positive and negative controls. qPCR assays were then performed to detect the ChIP DNA, and the fold enrichment was calculated as FE% = 2^(lgG CT‐Sample CT)^ × 100% according to the manufacturer's instructions. The sequences of the promoter‐specific primers are shown in Table S3.

### 4.9 Coimmunoprecipitation

Co‐IP assays were performed with a Pierce Classic IP kit (Thermo Fisher Scientific). According to the manufacturer's protocol, each IP assay required 1000 μg of total protein and 10 μg of affinity‐purified antibody. After incubation at 4°C overnight, the immune complex was gently captured with agarose by an end‐over‐end mixing for 2 h at 4°C. The immune complex was eluted and transferred to an SDS–PAGE gel for Western blotting with normal IgG as a negative control. The primary antibodies are provided in Table S2.

### 4.10 Corneal epithelial wound healing model

Animal studies were approved by the Animal Experiment Ethics Committee of Eye, Ear, Nose and Throat Hospital of Fudan University and were performed in accordance with the statement of the Association for Research in Vision and Ophthalmology (ARVO) entitled “Use of Animals in Ophthalmic and Vision Research.”

A corneal epithelial wound healing model was established in 8‐week‐old wild‐type (WT) C57BL/6 mice and Wnt16b knockout (KO) mice (B6/JGpt‐Wnt16b^em3Cd16769^/Gpt, GemPharmatech Co., Ltd, China) with a thorough scrape of the entire corneal epithelium under an operating microscope. Both WT mice and Wnt16b‐KO mice were randomly divided into a normal saline (NS)‐treated group and a WNT16b‐treated group. NS or 200 ng/mL rhWNT16b solution was topically applied three times a day from Day 0. The epithelial defect was evaluated under a slit lamp with fluorescence staining, and the percent epithelial defect area as compared to the original wound size was used to calculate the epithelial healing rate.

### 4.11 Establishment of LSCD model and hLESCs transplantation

C57/BL/6 mice (6–8‐weeks old male) were used to establish a unilateral LSCD model. After anaesthesia, the corneal and limbal epithelium of the left eyes were thoroughly scraped, followed by a cotton piece with 0.1 N sodium hydroxide (NaOH) covering on the entire limbal and corneal surface for 30 s. Then, the eye was flushed thoroughly with normal saline. After the injury, all eyes were treated with tobramycin and dexamethasone eyedrops topically three times a day for 2 weeks and underwent slip lamp examination and fluorescein staining two times a week until 60 days after injury. The degree of corneal epithelial defect and stromal neovascularization were graded as the following: score 0, none; score 1, area ≤ 1/4 cornea; score 2, 1/4 cornea < area ≤ 1/2 cornea; score 3, 1/2 cornea < area ≤ 3/4 cornea; score 4, area > 3/4 cornea. The degree of corneal opacity was evaluated as the following: score 0, none; score 1, mild, pupil clearly visible; score 2, moderate, pupil partially visible; score 3, severe, pupil faintly seen; score 4, severe, pupil not seen. The LSCD model was successfully established if epithelial defect score and corneal opacity score were more than two, and stromal neovascularization score was more than three.^41^, ^42^

The successful LSCD models were randomly assigned to three groups and treated with amniotic membrane (AM) transplantation without cultivated hLESCs (*n* = 6) (Non‐hLESCs group), routinely cultured LESCs and AM transplantation (*n* = 6) (Cntl‐LESCs group) and WNT16b co‐incubated hLESCs and AM transplantation (*n* = 6) (WNT16b‐hLESCs group). First, the conjunctivalized epithelium and 10–15 μm of anterior stroma with fibrovascular pannus were removed from the cornea. According to the grouping, 2 μL type I collagen suspension with WNT16b co‐incubated hLESCs (1 × 10^6^ cells/μL), routinely cultured LESCs (1 × 10^6^ cells/μL) or without hLESCs was evenly coated onto the cornea surface, then waited for several minutes until the collagen solution is solidified. Finally, a processed, hydrated, sterilized human amniotic membrane (Ruixiufu, Ruitai Biotech, Guangzhou, China) was fixated on the ocular surface with 10–0 suture to protect the transplanted hLESCs (Figure S4D). The sutures were removed 2 weeks after surgery. Then, 0.3% tobramycin and 0.1% dexamethasone were topically administrated four times a day for 10 weeks. All eyes underwent slip lamp examination and fluorescein staining at 0, 2, 4 and 10 weeks after the surgery. The entire corneas were harvested on 10 weeks for histopathology and immunostaining analysis.

### 4.12 Statistical analysis

Statistical analysis was performed using SPSS (version 22.0, SPSS, Inc., Chicago, Illinois, USA) and GraphPad Prism software (La Jolla, CA, Version 9). One‐way ANOVA or Student's *t* test was used for comparisons among multiple groups or between two groups. The *p* values less than 0.05 were considered statistically significant.

## AUTHOR CONTRIBUTIONS

Qihua Le and Lan Gong conceived and designed the study. Xichen Wan and Songjiao Zhao contributed to the design of the study and performed most of the experiments. Xichen Wan, Songjiao Zhao, Yiqin Dai and Jing Zhang collected and analysed the data. Xichen Wan and Songjiao Zhao wrote the draft. Qihua Le made critical revisions. All the authors approved the manuscript.

## CONFLICT OF INTEREST STATEMENT

The authors declare that they have no conflict of interest.

## Supporting information


**Data S1:** Supporting Information

## Data Availability

All data supporting the findings of this study are available within the paper and its supplementary information file.
