# Supplementary material for: WNT16b promotes the proliferation and self‐renewal of human limbal epithelial stem/progenitor cells via activating the calcium/calcineurin A/NFATC2 pathway
Source: Cell Prolif. 2023 Mar 27;56(10):e13460. doi: 10.1111/cpr.13460 (PMC10542615; doi:10.1111/cpr.13460)
Supplement: Supplementary file 1 — Data S1: Supporting Information [file CPR-56-e13460-s001.docx]

**Supplementary table 1: The specific primer sequences**

| **Gene** | **Primer** | **Sequences (5’-3’)** |
| --- | --- | --- |
| **Human** |  |  |
| PPP3CA | Forward | GCGCATCTTATGAAGGAGGGA |
| PPP3CA | Reverse | TGACTGGCGCATCAATATCCA |
| PPP3CB | Forward | CACCTGCATTTGGACCAATGT |
| PPP3CB | Reverse | AGGGAACCCTGTAGTTTGACTT |
| PPP3CC | Forward | ACCGCGTCATCAAAGCTGT |
| PPP3CC | Reverse | CTTCCAGTCGTCCTTCCTTTAC |
| PPP3R1 | Forward | CCTTTGGAAATGTGCTCACACT |
| PPP3R1 | Reverse | GGATTCTGTTGTAACTCAGGCAG |
| PPP3R2 | Forward | GCAGAAGTTGAGGTTTGCGTT |
| PPP3R2 | Reverse | TCTTGTGGATCTCCAGGTCTC |
| NFATC1 | Forward | CCCAGATGGCCACCATGT |
| NFATC1 | Reverse | AGGTCCCGGTCAGTTTTCG |
| NFATC2 | Forward | CATCTAACCCCATCGAGTGC |
| NFATC2 | Reverse | GCTGTCTGTGTCTTGTCTTTCAA |
| NFATC3 | Forward | TCCACCTCCATCTACTTTAACCA |
| NFATC3 | Reverse | TTGGGACCACCTAATGGGCT |
| NFATC4 | Forward | CATCCTACAGACCGGGCCT |
| NFATC4 | Reverse | CCTGTGGTACCCCTAGTCTCAGG |
| PRKCA | Forward | TGGGTCACTGCTCTATGGACTTATC |
| PRKCA | Reverse | CGCCCCCTCTTCTCAGTGT |
| CaMK2A | Forward | CATGGTTTGGGTTTGCAGGG |
| CaMK2A | Reverse | CCGGCTTTGATCTGCTGGTA |
| NLK | Forward | CCAACCTCCACACATTGACTATT |
| NLK | Reverse | ACTTTGACATGATCTGAGCTGAG |
| CDC42 | Forward | CCATCGGAATATGTACCGACTG |
| CDC42 | Reverse | CTCAGCGGTCGTAATCTGTCA |
| ACTB | Forward | GTGGCCGAGGACTTTGATTG |
| ACTB | Reverse | CCTGTAACAACGCATCTCATATT |
| **Mouse** |  |  |
| Ppp3ca | Forward | GAGCCCAAGGCGATTGATCC |
| Ppp3ca | Reverse | ATCCACACGAGGTTTCCCATC |
| Nfatc2 | Forward | CCACCACGAGCTATGAGAAGA |
| Nfatc2 | Reverse | GTCAGCGTTTCGGAGCTTCA |
| Actb | Forward | GGCTGTATTCCCCTCCATCG |
| Actb | Reverse | CCAGTTGGTAACAATGCCATGT |
| Wnt16 | Forward | CAGGGCAACTGGATGTGGTT |
| Wnt16 | Reverse | CTCGTGTCGGAACTGGCTTC |
| Myc | Forward | CCCTATTTCATCTGCGACGAG |
| Myc | Reverse | GAGAAGGACGTAGCGACCG |
| Foxm1 | Forward | CAGAATGCCCCGAGTGAAACA |
| Foxm1 | Reverse | GTGGGGTGGTTGATAATCTTGAT |

**Supplementary table 2: the primary antibodies and the dilutions**

| **Primary antibody** | **Manufacturer** | **Dilution** | **Application** |
| --- | --- | --- | --- |
| anti-Calcineurin A (pan) antibody | Cell Signaling Technology | 1:1000 | WB |
| anti-NFAT1 antibody | Cell Signaling Technology | 1:250 | WB |
| anti- NFATC1 antibody | Invitrogen | 1:250 | WB |
| anti-cdc42 antibody | Abcam | 1:1000 | WB |
| anti-Phospho-PKC (pan) antibody | Cell Signaling Technology | 1:1000 | WB |
| anti-CaMKⅡ antibody | Cell Signaling Technology | 1:1000 | WB |
| anti-Phospho-CaMKⅡ antibody | Cell Signaling Technology | 1:1000 | WB |
| anti-PLCβ3 antibody | Cell Signaling Technology | 1:1000 | WB |
| anti-Phospho-PLCβ3 antibody | Abcam | 1:1000 | WB |
| anti-β-actin antibody | Cell Signaling Technology | 1:1000 | WB |
| anti-Histone 3 antibody | Cell Signaling Technology | 1:1000 | WB |
| anti-HDAC3 antibody | Cell Signaling Technology | 1:1000 | CO-IP |
| anti-WDR5 antibody | Cell Signaling Technology | 1:1000 | CO-IP |
| anti-GCN5L2 antibody | Cell Signaling Technology | 1:1000 | CO-IP |
| anti-P300 antibody | Cell Signaling Technology | 1:1000 | CO-IP |
| anti-EZH2 antibody | Cell Signaling Technology | 1:1000 | CO-IP |
| anti-Suv39H1 antibody | Cell Signaling Technology | 1:1000 | CO-IP |
| anti-HDAC1 antibody | Abcam | 1:1000 | CO-IP |
| anti-HDAC2 antibody | Cell Signaling Technology | 1:1000 | CO-IP |
| anti-NFAT1 antibody | Cell Signaling Technology | 1:50 | IF |
| anti-P63α antibody | Abcam | 1:200 | IF |
| anti-Ki67 antibody | Santa-Cruz Biotechnology | 1:50 | IF |

**Supplementary table 3: The sequence and primers of ChIP-qPCR assay**

| **FoxM1: chr12: 2877495-2877914**  ***Sequence:***  GCCACTATGCCCAGCCCACATTTGTTTATTTGATTAAAATGTCTGTGCCCCTCTTCCAGG  ATTGGGCTGTGAGCCCAGGGGAAGGAAAGAACCTTGTCTGCCATTGTATCTTCAGGGCCT  AGCGGTGCCTGGCGCACAGCAGTTGCTCAACTAGACTGGTTGACTAAGTCAATAAATAAA  GCACTACGGTCTATTATATCCGAAGGCTTGGCTTCGGGAGGGGCAAAAGACAGGTTTCGC  GCTGAGGTAGGGTTCATGGTGCCGACATTTTTTTTCAAGATGGAAGAAAGCGGAGATAAT  ACGCAGCCCTCAAAGGAACTTAGTCTAATCGGGGGGAGCAGACGATCGTTCACTGTGGGA  AAATGGGGTACGATTTCCCCCAGTGAGGAAATCAACTAAAGCCGAGCTTTGAAAAGGGGA  ***Primer:* Forward**: GCCCAGCCCACATTTGTTTA  **Reverse**: CGCTAGGCCCTGAAGATACA |
| --- |
| **MYC: chr8: 127734711-127735070**  ***Sequence:***  AAATTAATGCCTGGAAGGCAGCCAAATTTTAATTAGCTCAAGACTCCCCCCCCCCCAAAA  AAAGGCACGGAAGTAATACTCCTCTCCTCTTCTTTGATCAGAATCGATGCATTTTTTGTG  CATGACCGCATTTCCAATAATAAAAGGGGAAAGAGGACCTGGAAAGGAATTAAACGTCCG  GTTTGTCCGGGGAGGAAAGAGTTAACGGTTTTTTTCACAAGGGTCTCTGCTGACTCCCCC  GGCTCGGTCCACAAGCTCTCCACTTGCCCCTTTTAGGAAGTCCGGTCCCGCGGTTCGGGT  ACCCCCTGCCCCTCCCATATTCTCCCGTCTAGCACCTTTGATTTCTCCCAAACCCGGCAG  ***Primer*:** **Forward**: GGGGAAAGAGGACCTGGAAA  **Reverse:** TCAGCAGAGACCCTTGTGAA |

**Supplementary figure 1**


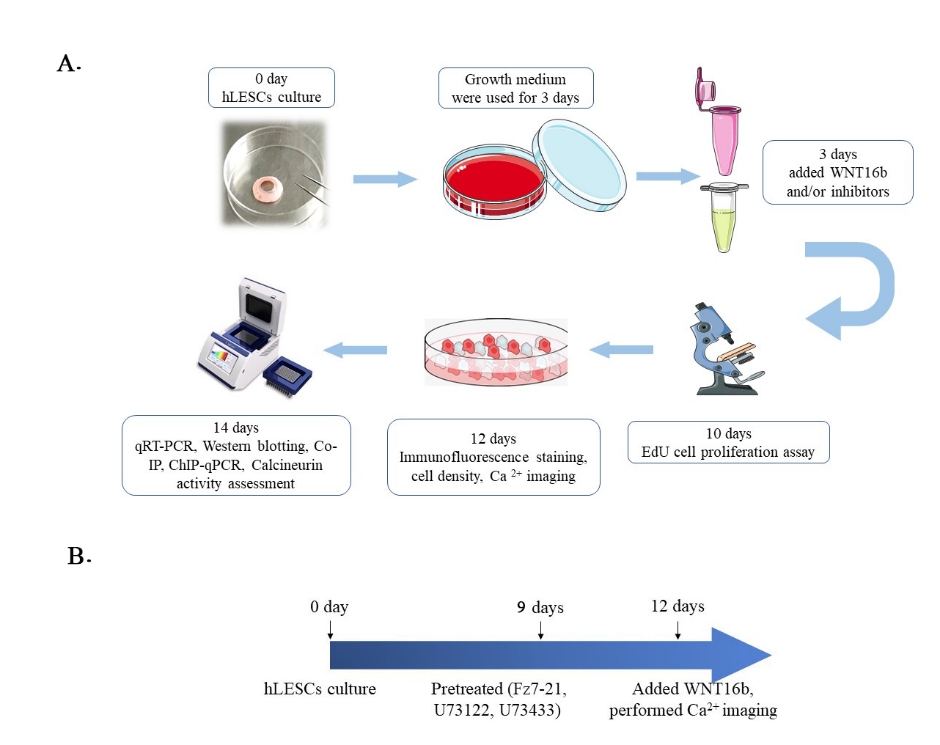


**Figure legend**

The schematic diagram of the experiment methods.

Primary human limbal epithelial cells were cultured and after growing in growth medium for 3 days, recombinant human WNT16b and/or other inhibitors including FK506, VIVIT and Fz7-21 were added to regulate hLESCs. After 10 days treatment, EdU cell proliferation assay was detected and Immunofluorescence staining, cell density and Ca^2+^ imaging were performed in day 12. After 14 days, other methods including qRT-PCR, Western blotting, Co-IP, ChIP-qPCR and calcineurin activity assessment were used. (Supplementary figure 3A)

For intracellular calcium imaging detecting, hLESCs were cultured in growth medium for 9 days, then some specific inhibitors were pretreated for 3 days. In 12 days, Ca^2+^ imaging was performed.

**Supplementary figure 2**

**
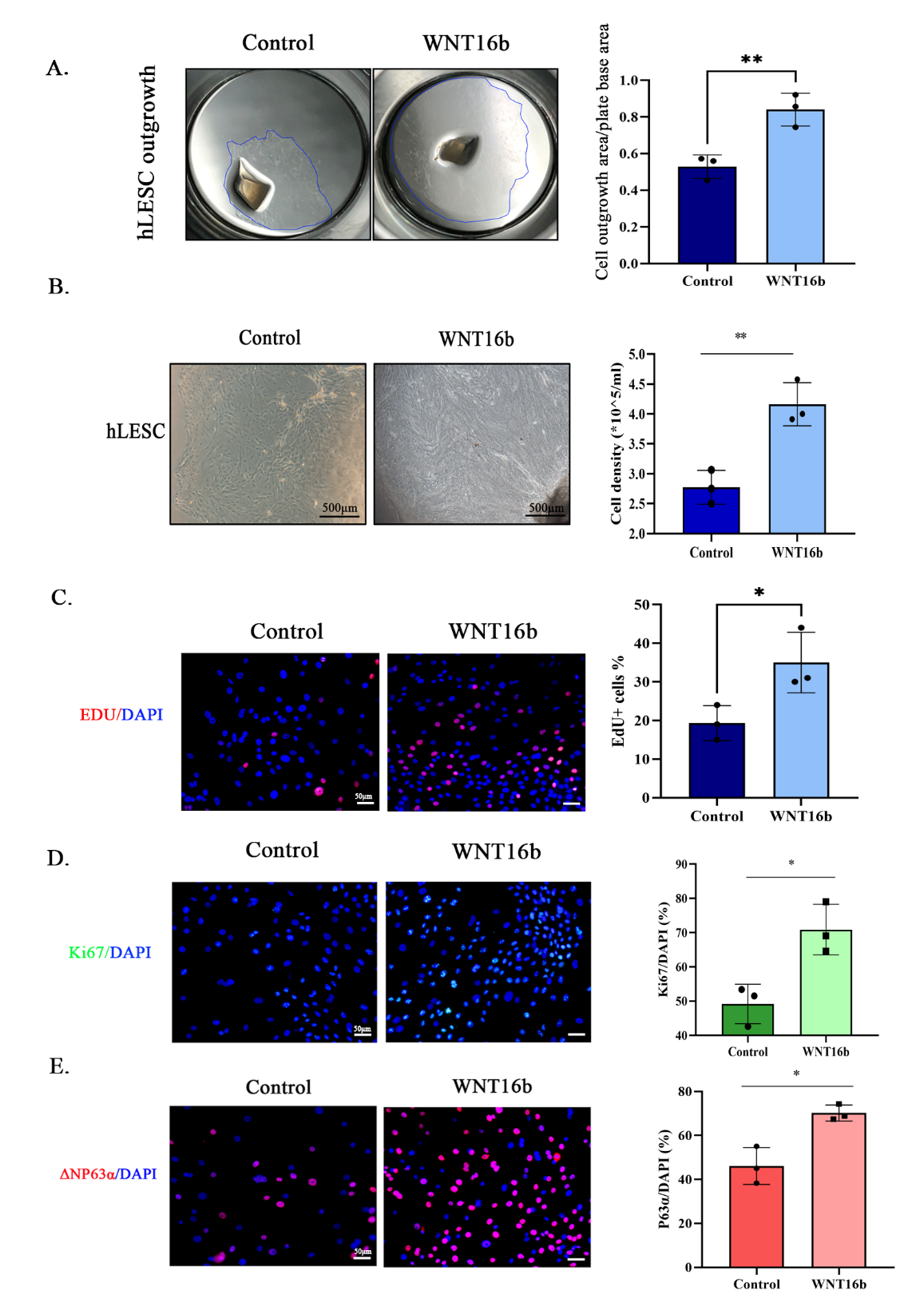
**

**Figure legend**

Compared with control group, the treatment of 200 ng/ml WNT16b promoted cell growth, which is shown as a larger cell outgrowth area (Supplementary figure 2A) and a higher cell density (Supplementary figure 2B). Moreover, a higher percentage of EdU+, Ki67+ and ΔNP63α+ cells in WNT16b group indicates that WNT16b is crucial for maintaining the stemness of hLESCs. (Supplementary figure 2C-E), Scar bar: 50 μm (C-E).

**Supplementary figure 3**


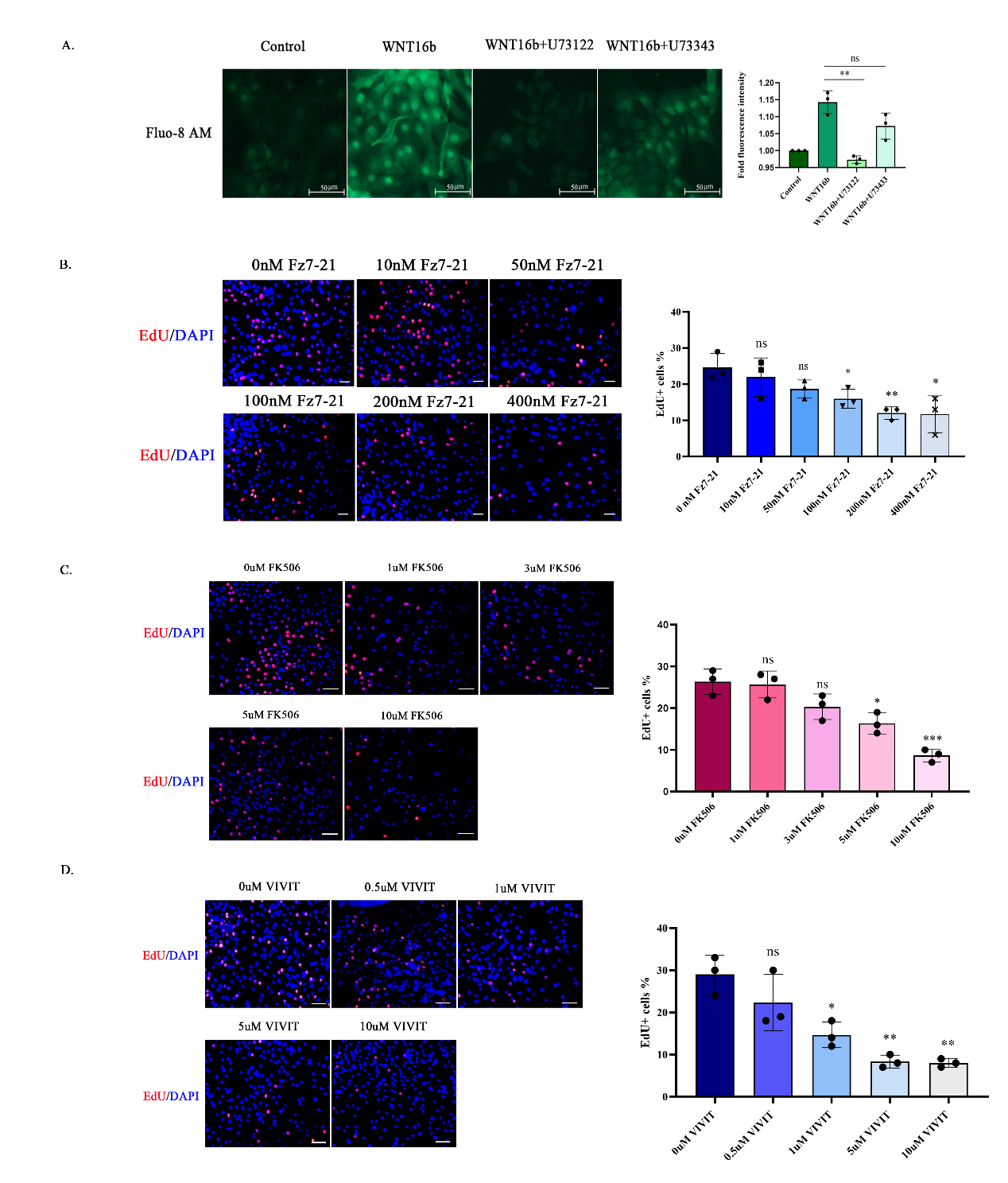


**Figure legend**

WNT16b can promote the intracellular calcium release, but U73122, the inhibitor of PLCβ3, reduced this process. Its inactive analogue U73343 (negative control of U73122) had no such effect. (Supplementary figure 3A)

EdU assay in different concentrations of Fz7-21, FK506 and VIVIT treated were performed to evaluate the proliferative effect of Fz7-21, FK506 and VIVIT (Supplementary figure 3B-D), 200nM Fz7-21, 5uM FK506 and 1uM VIVIT were used for further experiment. Scar bar: 50 μm (B), 100 μm (C,D) .

**Supplementary figure 4**


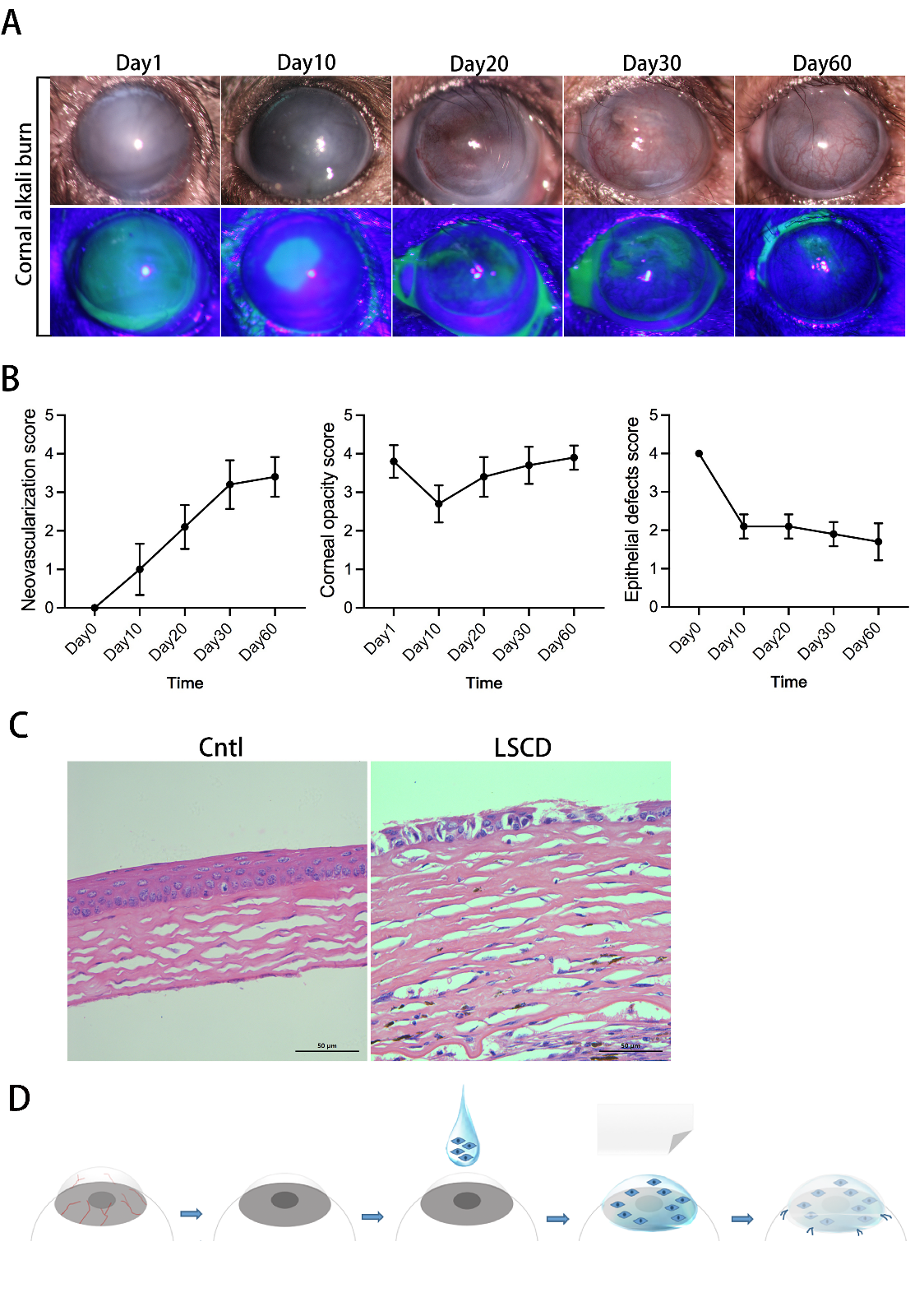


**Figure legend**

Evaluation of mouse ocular surface after alkali burn.

Representative images of ocular surface on 1, 10, 20, 30 and 60 days. (Supplementary figure 4A) Neovascularization developed dramatically during 30 days after alkali burn. Corneal opacity was improved transiently during 10 days after injury but developed subsequently. Epithelial defect partially recovered during 10 days after injury but existed persistently. (Supplementary figure 4B) The histopathology of cornea from normal and LSCD mice. The corneal epithelial layers were disorganized and significantly decreased in LSCD eyes. The corneal stroma was dramatically thickening with neovascularization and inflammatory infiltration in LSCD eyes. (Supplementary figure 4C)
